# Electronic Chip Package and Co-Packaged Optics (CPO) Technology for Modern AI Era: A Review

**DOI:** 10.3390/mi16040431

**Published:** 2025-04-02

**Authors:** Guoliang Chen, Guiqi Wang, Zhenzhen Wang, Lijun Wang

**Affiliations:** Hangzhou Institute of Technology, Xidian University, Hangzhou 311231, China; chenglchengl@sina.com (G.C.); wgq05253537@163.com (G.W.); wangzhenzhen@xidian.edu.cn (Z.W.)

**Keywords:** semiconductor conventional and advanced package, fan-out, 2D, 2.5D and 3D package, interposer, chiplets, co-packaged optics, silicon and glass, femtosecond laser

## Abstract

With the growing demand for high-performance computing (HPC), artificial intelligence (AI), and data communication and storage, new chip technologies have emerged, following Moore’s Law, over the past few decades. As we enter the post-Moore era, transistor dimensions are approaching their physical limits. Advanced packaging technologies, such as 3D chiplets hetero-integration and co-packaged optics (CPO), have become crucial for further improving system performance. Currently, most solutions rely on silicon-based technologies, which alleviate some challenges but still face issues such as warpage, bumps’ reliability, through-silicon vias’ (TSVs) and redistribution layers’ (RDLs) reliability, and thermal dissipation, etc. Glass, with its superior mechanical, thermal, electrical, and optical properties, is emerging as a promising material to address these challenges, particularly with the development of femtosecond laser technology. This paper discusses the evolution of both conventional and advanced packaging technologies and outlines future directions for design, fabrication, and packaging using glass substrates and femtosecond laser processing.

## 1. Introduction

**Figure 1:**
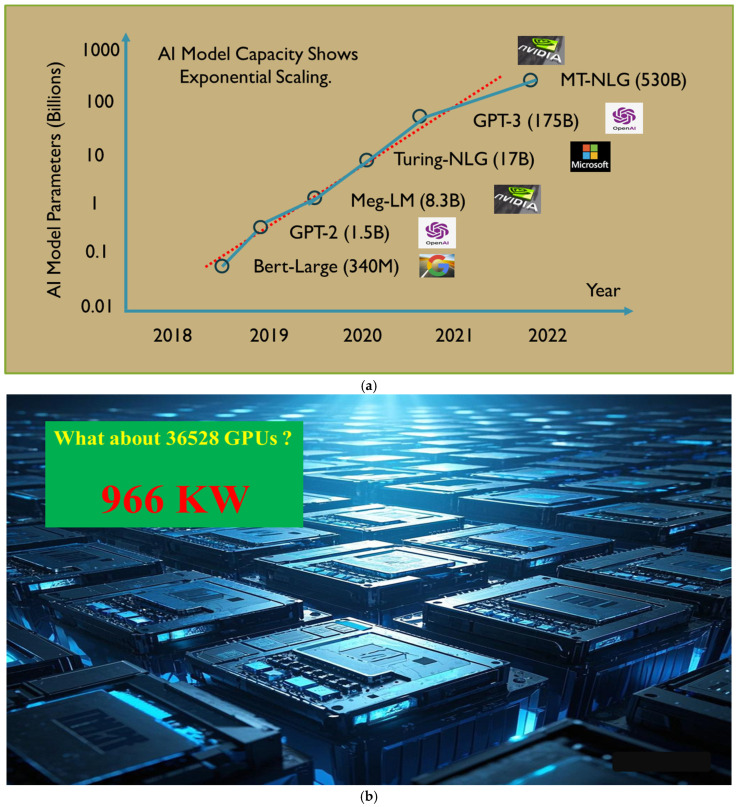
AI computing power demand and energy consumption. (**a**) Exponentially increasing demand for data computation in AI. (**b**) Huge energy consumption in data center GPU clusters.

The challenges in modern HPC, AI, and data communication systems are becoming increasingly prominent, especially in hyperscale data centers where tens of thousands of CPU and GPU clusters are deployed, and data transfer rates are growing exponentially. From a system perspective, key considerations today include speed, bandwidth, power efficiency, and cost, as illustrated in Figure 1, which is based on data from OpenAI and Broadcom’s official reports [1,2].

**Figure 2:**
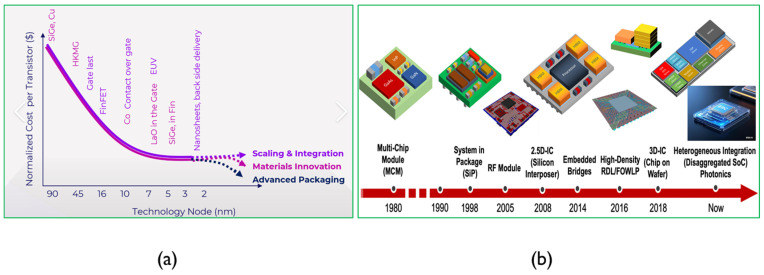
Moore’s Law and advanced package. (**a**) Moore’s Law scaling down to its physical limit; adapted from A Century of Moore’s Law—SemiAnalysis. (**b**) Advanced package roadmap to 3D chiplets and CPO.

To address these challenges, several approaches have been proposed including scaling down transistor feature sizes, exploring novel materials and devices, leveraging quantum information technologies, etc. As transistor sizes approach their physical limits, advanced packaging technologies have emerged as an effective alternative with rapid developments in 3D chiplets hetero-integration and co-packaged optics (CPO) [3,4,5,6,7,8], as shown in Figure 2, adapted from semiconductor-related websites. Currently, most of these technologies are based on silicon, which is a pretty common material in the semiconductor industry. While successful to some extent, challenges still remain including opto-electronic co-design, wafer warpage, reliability, and power consumption [9,10,11,12]. Recently, glass materials have attracted significant attention due to their excellent mechanical, thermal, electrical, and optical properties, which address many of these challenges. Many fabrication methods are available for glass, with femtosecond laser technology emerging as a promising technique. While glass has been applied in various fields, its use in advanced packaging remains limited, particularly when combined with femtosecond laser fabrication technology [13,14,15,16]. This paper aims to analyze the evolution of packaging technologies and propose future directions for work in this area.

## 2. Conventional Packaging Technology

**Figure 3:**
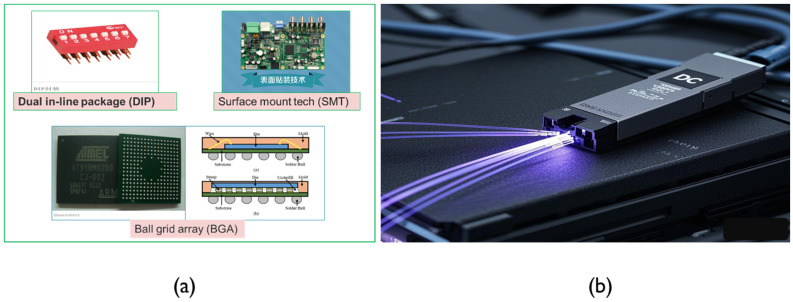
(**a**) Conventional electronic package. Adapted from IC Package Types | DIP, SMT, BGA, FC. (**b**) Conventional optical module.

Conventional electronic and opto-electronic packaging technologies primarily refer to the period before the 21st century. During this time, mainstream technologies included dual in-line package (DIP), surface mount technology (SMT), ball grid array (BGA), flip-chip (FC), and optical module, as shown in Figure 3. These technologies gradually improved integration density and overall performance with each generation as shown in Table 1.

### 2.1. Dual In-Line Package (DIP)

A Dual In-Line Package (DIP) is a type of electronic component package commonly used for integrated circuits (ICs) and other electronic devices. It features a rectangular shape with two parallel rows of pins (typically ranging from 4 to 64 pins) that extend from both sides of the package, allowing it to be mounted onto a printed circuit board (PCB) through-hole technology. DIPs are widely used for their ease of handling, soldering, and replacement, making them a popular choice in prototyping and low-volume manufacturing. While they have largely been replaced by smaller, more compact packages in modern devices, DIPs remain valued for their durability and straightforward mechanical design in certain applications [17,18,19].

### 2.2. Surface Mount Technology (SMT)

Surface Mount Technology (SMT) is a method of electronic assembly where components are directly mounted onto the surface of a printed circuit board (PCB), rather than being inserted into holes as in traditional through-hole technology. SMT allows for smaller, more compact designs due to the smaller size of the components and the ability to place them on both sides of the PCB. This technology enables faster production speeds, improved reliability, and greater automation in manufacturing, making it a dominant choice in modern electronics. The process typically involves soldering components using automated machines like pick-and-place machines, followed by reflow soldering to secure the components in place. SMT has significantly reduced the size and cost of electronic devices while enhancing performance [20,21,22].

SMT can be further classified into the following four categories:

#### 2.2.1. SOP (Small Outline Package)

A Small Outline Package (SOP) is a type of surface-mounted package that features a rectangular or square body with leads (pins) extending from both sides. These leads are bent at right angles and are soldered onto the PCB (Printed Circuit Board) surface [23,24].

#### 2.2.2. QFN (Quad Flat No-Lead)

A Quad Flat No-lead (QFN) package is a surface-mounted component with a square or rectangular body and leads on all four sides. Unlike traditional packages, QFN components have no leads extending from the sides; instead, they feature pads on the bottom that directly connect to the PCB via soldering [25,26].

#### 2.2.3. QFP (Quad Flat Package)

A Quad Flat Package (QFP) is similar to the SOP but with a larger form factor and more pins. It is a square or rectangular package with leads extending from all four sides, which are soldered to the PCB in a surface-mounted fashion [27,28].

#### 2.2.4. SOT (Small Outline Transistor)

A Small Outline Transistor (SOT) package is one of the smallest surface-mounted packages, usually used for transistors and diodes. It has leads that extend from two opposite sides of the package, allowing for surface mounting on the PCB [29,30].

### 2.3. Ball Grid Array (BGA)

A Ball Grid Array (BGA) is a type of surface-mounted packaging for integrated circuits (ICs) where the connections to the PCB are made via an array of small solder balls arranged in a grid pattern on the underside of the package. Unlike traditional packages with pins, BGAs offer improved electrical performance, higher density, and better thermal dissipation due to their direct connection to the PCB. The solder balls provide both mechanical support and electrical connectivity, allowing for more compact and reliable designs, especially for high-performance chips like processors and memory. BGAs are widely used in applications requiring high pin counts and are typically soldered to the PCB using reflow soldering techniques [31,32,33].

### 2.4. Flip-Chip (FC)

Flip-Chip (FC) packaging is an advanced method of mounting integrated circuits (ICs) directly onto a printed circuit board (PCB) by flipping the chip upside down and connecting it to the board using tiny solder bumps or micro-bumps. This eliminates the need for traditional wire bonding, allowing for a more compact and efficient design with improved electrical performance and faster signal transmission. Flip-chip technology offers superior heat dissipation and reduced signal path lengths, making it ideal for high-speed and high-performance applications like processors, graphics chips, and memory devices. Additionally, FC packages can be used in applications that require dense interconnects and are well-suited for multi-chip modules or system on chip (SoC) designs [34,35,36].

### 2.5. Optical Module

In the optical communication domain, traditional packaging technologies such as the optical module shown in Figure 3b have dominated the market for decades [37,38,39]. An optical module is a key component in optical communication systems that facilitates the conversion between electrical and optical signals, enabling high-speed data transmission over fiber-optic networks. It typically consists of a light-emitting device, such as a laser diode or vertical-cavity surface-emitting laser (VCSEL), and a light-detecting device, such as a photodiode, along with the necessary driver and receiver electronics. Optical modules are used in various networking applications, including data centers, telecommunications, and high-speed internet connections, where they provide reliable, long-distance signal transmission with minimal loss and electromagnetic interference. These modules are commonly available in different form factors, such as SFP (Small Form-factor Pluggable), QSFP (Quad Small Form-factor Pluggable), and CFP (C Form-factor Pluggable), depending on the specific requirements of the communication system.

## 3. Advanced Packaging Technology

In the 21st century, numerous advanced packaging technologies have been developed, including fan-out (FO), 2D, 2.5D, and 3D integration, which feature key elements such as redistribution layers (RDLs), silicon or glass-based interposers, through-silicon vias (TSVs), and through-glass vias (TGVs). Meanwhile, the optical module, enabled by silicon photonics, is now treated similarly to electronic chips, and advanced co-packaged optics (CPO) is being extensively researched and developed.

### 3.1. Fan-Out (FO) Package

A Fan-Out package is an advanced semiconductor packaging technology where the integrated circuit (IC) is mounted on a die with a larger area, and the electrical connections are redistributed from the die’s I/O pads to external solder balls arranged around the perimeter of the package, creating a “fan-out” pattern. This approach allows for a higher number of input/output (I/O) connections while maintaining a smaller, more compact form factor compared to traditional packages. Fan-Out packages are particularly beneficial for applications requiring high-density interconnections, such as mobile devices, high-performance processors, and System-in-Package (SiP) designs. By using techniques like wafer-level packaging (WLP), FO packages offer enhanced thermal performance, improved signal integrity, and better electrical performance, making them a preferred choice in modern, high-speed, and miniaturized electronic systems.

**Figure 4:**
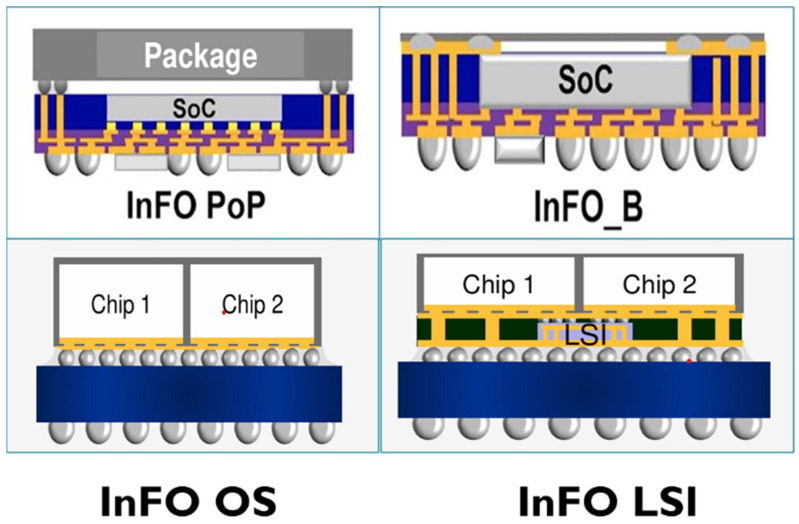
Schematic diagram of TSMC integrated Fan-out (InFO) package [40].

TSMC’s Integrated Fan-Out (InFO) is a representative FO package, as shown in Figure 4. This technology directly uses RDLs and Through InFO Via (TIV) to connect chips to the PCB, significantly reducing parasitic parameters associated with bonding wires, thus enhancing overall performance and lowering costs. InFO is widely applied in high-performance computing, AI, mobile devices, and other fields.

TSMC’s InFO technology has been used in Apple’s A-series processors and DRAM co-packages. In this configuration, the top bumps of the DRAM are connected to the RDL via TIV, which in turn connects to the logic chip. This design reduces the PCB area required while maintaining excellent thermal and electrical performance. Several derivatives of InFO, such as InFO PoP, InFO B, InFO OS, InFO LSI, InFO MS, and InFO AiP, have been developed since then [40,41,42].

InFO PoP (Integrated Fan-Out Package-on-Package) is a 3D packaging solution that combines TSMC’s fan-out wafer-level packaging (FOWLP) technology with a stacked package-on-package (PoP) design. InFO PoP integrates multiple chips vertically within a single package to save space and enhance performance. It is typically used for high-performance mobile devices and servers [43,44].

InFO B (InFO Bump) is a variation of the InFO packaging technology designed to further improve the performance of semiconductor devices by integrating a bumping process within the fan-out structure. The primary difference between InFO_B and traditional InFO packaging is the addition of bumps on the substrate to create a more efficient electrical connection between the chip and the board [45,46].

InFO OS (InFO Open Substrate) is an advanced packaging technology that focuses on integrating the fan-out structure with an open substrate. Unlike traditional fan-out packages, InFO OS features a larger die area with the fan-out structure, allowing for more versatility and adaptability [47,48].

InFO LSI (InFO Logic System Integration) is a packaging solution focused on integrating logic and memory functions on a single chip or in a single package. It enables the combination of high-performance logic dies with memory dies, providing improved performance and reduced power consumption [49,50].

InFO MS (InFO Multi-Stack) is an advanced 3D packaging solution from TSMC that allows the stacking of multiple chips in a single package, offering a compact and high-performance solution for demanding applications. It integrates fan-out packaging with vertical stacking of multiple dies [51,52].

InFO AiP (InFO Antenna-in-Package) is a specialized packaging solution that integrates antennas within the package. This technology is particularly beneficial for mobile devices and applications where antennas need to be part of the integrated system in a compact form [53,54].

### 3.2. Advanced 2D Packaging

An advanced 2D semiconductor package is a cutting-edge technology used to enhance the performance, density, and efficiency of integrated circuits (ICs). In a 2D packaging structure, multiple semiconductor devices, such as chips or dies, are placed on a single substrate in a flat, two-dimensional arrangement, which contrasts with 3D packaging, where chips are stacked vertically. This packaging approach offers several advantages, including improved thermal management, reduced signal delay, and increased interconnect density, all while maintaining a relatively simple manufacturing process. Typically, the chips are connected through fine-pitch interconnections, using wire bonding, flip-chip technology, or other advanced bonding techniques like micro-bumps, ensuring high-speed communication between the devices.

**Figure 5:**
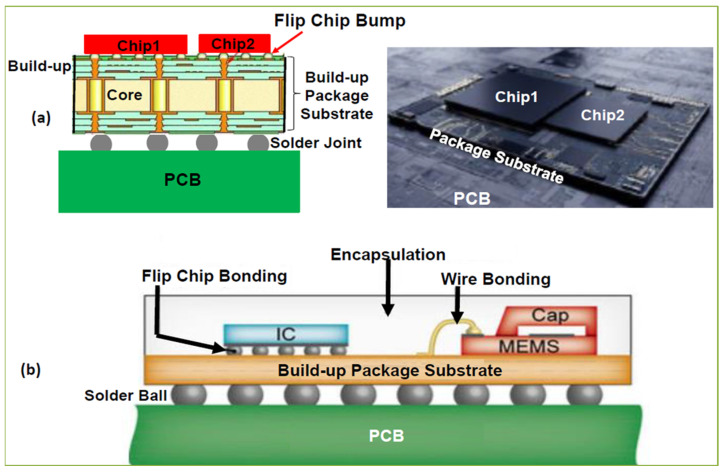
Schematic diagram of typical advanced 2D package for chiplets [55].

Advanced 2D packages often incorporate features like system-in-package (SiP) integration, where various components like processors, memory, and power management circuits are combined into one compact package. This integration enables smaller form factors while improving overall system performance as shown in the Figure 5. Additionally, 2D packages are often optimized for high-frequency applications, such as 5G communication systems and AI hardware, where speed and reliability are critical. They also find use in consumer electronics, automotive, and industrial applications, where compactness, energy efficiency, and cost-effectiveness are highly valued [55,56,57,58,59,60].

**Figure 6:**
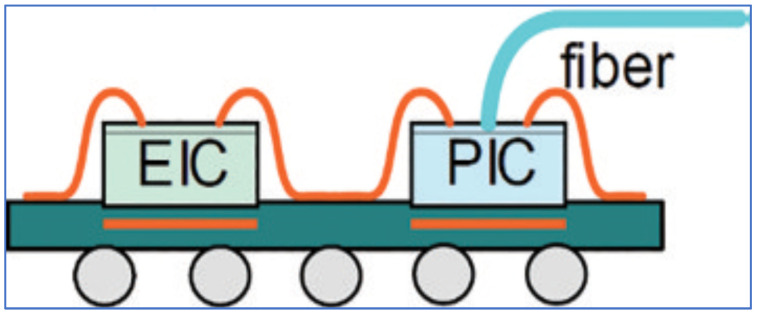
Schematic diagram of TSMC’s advanced COUPE package for 2D CPO [61].

Another typical example is provided here for a 2D CPO application. Figure 6 shows Compact Optical Universal Photonic Engine (COUPE) designed by TSMC in 2021 [61]. COUPE is a groundbreaking technology designed to address the growing demand for high-performance computing, networking, and communication systems, particularly in the fields of data centers and artificial intelligence.

This technology combines advanced photonic and semiconductor integration, offering a more compact, energy-efficient solution for optical interconnects. COUPE uses integrated photonic components that enable faster data transmission with lower power consumption compared to traditional electronic solutions, making it a key enabler for the next generation of high-speed, high-bandwidth applications. By providing enhanced performance in a small form factor, COUPE allows for more efficient, scalable, and faster communication between chips, which is essential for handling the increasing complexity and data traffic in modern computing systems. It marks a significant step in TSMC’s efforts to innovate and lead in the development of cutting-edge semiconductor technologies [62,63,64].

### 3.3. Silicon or Glass 2.5D Packaging

A 2.5D package is a type of advanced semiconductor packaging that involves placing multiple integrated circuit (IC) chips on a single interposer, which serves as a platform to connect the chips via high-density interconnects. Unlike traditional 2D packaging, where components are stacked or placed side by side without direct communication, 2.5D packaging allows for closer proximity between chips, enabling faster data transfer between them through the interposer’s dense wiring. This technology is commonly used in high-performance computing, networking, and graphics processing applications, as it offers a balance between the performance advantages of 3D stacking (such as reduced signal delay) and the manufacturing simplicity of 2D packaging. The 2.5D packages also provide improved power efficiency, thermal dissipation, and scalability for multi-chip systems, making them ideal for applications like high-end servers, AI processors, and system-in-package (SiP) designs. The 2.5D packaging approach uses interposers to connect multiple chips, including silicon photonics chips, effectively increasing interconnection density, reducing chip area, and lowering costs while enhancing performance metrics like computational efficiency and communication bandwidth. Both silicon and glass interposers are viable for these applications, with a silicon interposer infrastructure already well established, while glass-based interposers are progressing [65,66,67].

**Figure 7:**
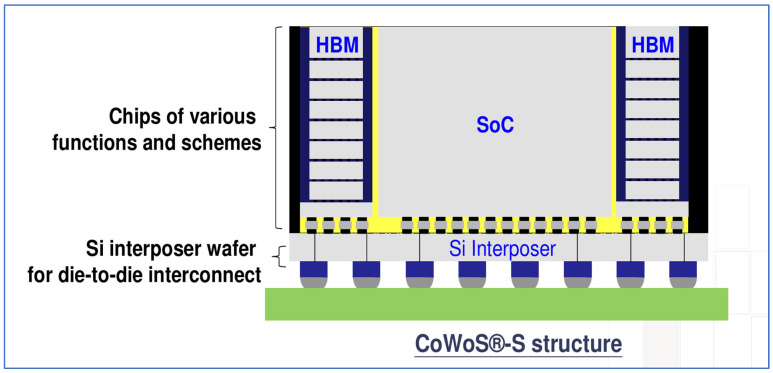
Schematic diagram of typical TSMC Chip-on-Wafer-on-Substrate (CoWoS) package [71].

**Figure 8:**
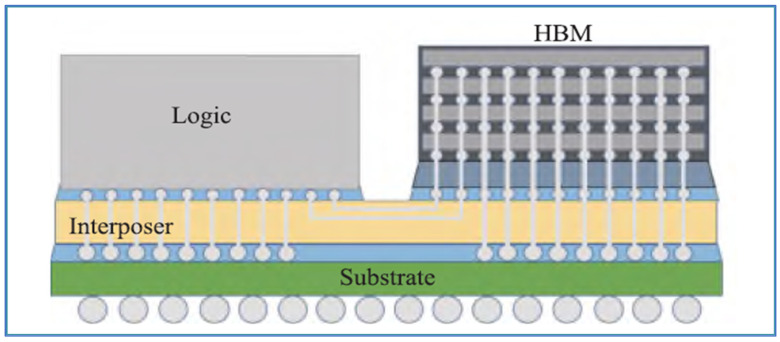
Schematic diagram of Samsung I-Cube4 package [71].

Some typical examples are provided for a silicon-based 2.5D chiplets package. One is TSMC’s Chip-on-Wafer-on-Substrate (CoWoS) as shown in Figure 7. Multiple dies with different functions are interconnected by RDLs and TSVs in the silicon interposer, enabling ultra-high-density, high-performance packaging. This is particularly suitable for AI and supercomputing applications [68,69,70,71]. The other is Samsung’s Interposer Cube 4 (I-Cube4) as shown in Figure 8. I-Cube4 is an advanced 2.5D silicon interposer packaging technology that utilizes stacking to integrate multiple components, such as memory and logic chips, into a compact and high-performance package. As the fourth generation of Samsung’s I-Cube (Integrated-Cube) technology, I-Cube4 offers improved bandwidth, reduced latency, and energy-efficient performance, making it ideal for high-performance computing, AI, and data-intensive applications. By shortening the distance for data transfer between stacked chips, I-Cube4 enables faster processing and more efficient power consumption, supporting the demands of modern data centers, AI accelerators, and other cutting-edge computing systems [72,73,74].

TSMC’s CoWoS-S technology and Samsung’s I-Cube4 technology both represent advanced packaging solutions, but with distinct approaches to enhancing performance and integration. TSMC’s CoWoS-S integrates multiple chips in a 2.5D configuration, utilizing a silicon interposer that connects high-bandwidth memory (HBM) to logic chips, offering high-performance computing with low latency and increased bandwidth. The technology supports heterogeneous integration, enabling the combination of different chip types such as CPU, GPU, and memory in a compact form. CoWoS-S also emphasizes high-density interconnects and efficient heat dissipation for large-scale data center and AI applications. In contrast, Samsung’s I-Cube4 builds on its previous I-Cube technologies and focuses on stacking logic and memory chips with an advanced interposer, enabling higher integration densities and faster data transfer speeds. The I-Cube4 also incorporates HBM with logic, but its interposer design supports an even higher level of customization and chip integration. While both technologies target similar markets such as high-performance computing and AI, Samsung’s I-Cube4 is designed to support even higher integration density and bandwidth, positioning it for cutting-edge applications requiring extreme performance, like AI accelerators and networking systems. Both innovations push the envelope in 2.5D packaging, but Samsung’s solution tends to emphasize greater interconnect scalability.

Another case is co-packaged optics (CPO) in which Photonic Integrated Circuits (PICs) and Electronic Integrated Circuits (EICs) are interconnected by a silicon or glass interposer. The 2.5D CPO technique is an innovative packaging technology that integrates optical and electronic components within a single package to enhance the performance of high-speed, high-bandwidth systems such as data centers and telecommunications infrastructure. In a CPO design, optical transceivers, including light sources (lasers) and detectors, are placed on the same silicon interposer alongside the electronic chips, forming a tightly integrated system. This integration reduces the need for external optical-to-electrical conversions, significantly improving power efficiency, signal integrity, and overall system bandwidth. CPO leverages the benefits of 2.5D packaging, where the optical and electronic components communicate through high-density interconnects on the interposer, minimizing the length and complexity of the signal paths. As a result, CPO is ideal for addressing the challenges of scaling data transmission speeds and power consumption in modern high-performance computing environments, offering a promising solution for the growing demands of optical interconnects in hyperscale networks.

**Figure 9:**
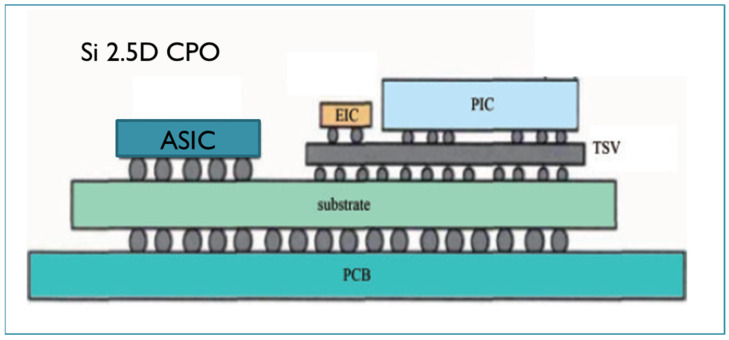
Typical 2.5D silicon-based CPO structure by IME of Chinese Academy [61].

**Figure 10:**
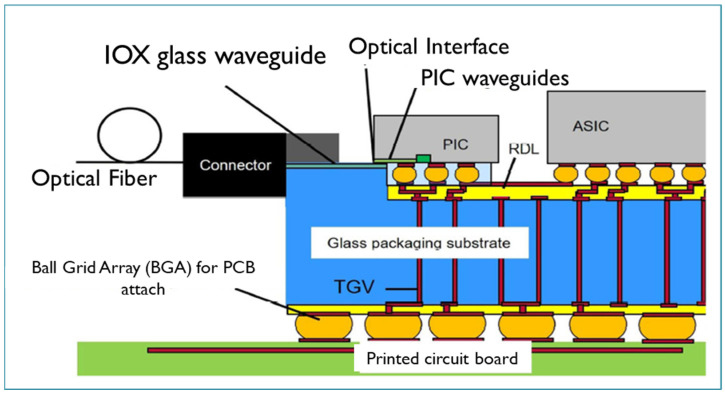
Typical 2.5D glass-based CPO structure by Corning, USA [76].

Some typical examples are provided here for both silicon and glass 2.5D CPO. Figure 9 shows a silicon-based CPO designed by IME of Chinese Academy. In this package, the PIC and EIC are connected by TSVs and three RDLs. The RDL line width and space can be less than 10 um, so a super-high-density interconnection and much smaller package dimensions can be implemented [61]. Another example is glass substrate CPO designed by Corning, USA, as shown in Figure 10. In the design, chiplets are connected by fine-line electrical copper routing inside a single-sided etched cavity. The PIC is connected to optical fibers by the glass waveguides fabricated with ion exchange (IOX) technology. These enable significant enhancement of the overall performances [75,76].

### 3.4. Silicon or Glass 3D Packaging

To further improve the whole chip system performances, 3D integration has been developed. A 3D package is an advanced semiconductor packaging technique where multiple integrated circuit (IC) chips are stacked vertically in a single package, with the chips connected through vertical interconnects, such as Through-Silicon Vias (TSVs), Through-Glass Vias (TGVs), or micro-bumps. This vertical stacking allows for a significant reduction in footprint while improving performance by shortening the distance for signal transmission between chips, leading to faster data exchange and lower power consumption. This 3D packaging is ideal for applications that require high performance, such as processors, memory, and high-bandwidth data systems, because it offers increased integration density, reduced latency, and better thermal management compared to traditional 2D packaging. Additionally, 3D packages can combine different types of ICs, such as logic, memory, and power management, into a single compact unit, enabling more efficient and powerful system designs. This technology is particularly advantageous in fields like high-performance computing, artificial intelligence, and mobile devices, where size, speed, and energy efficiency are crucial [77,78,79,80,81,82].

**Figure 11:**
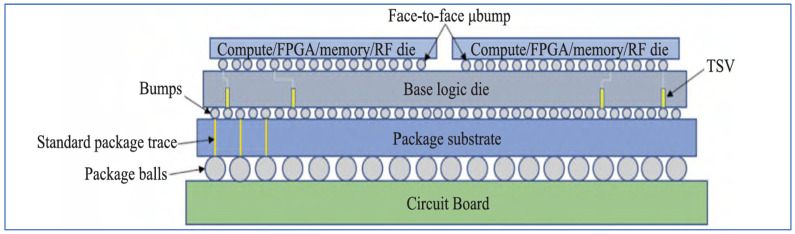
Typical 3D silicon-based chiplet hetero-integration by Intel, USA [71].

**Figure 12:**
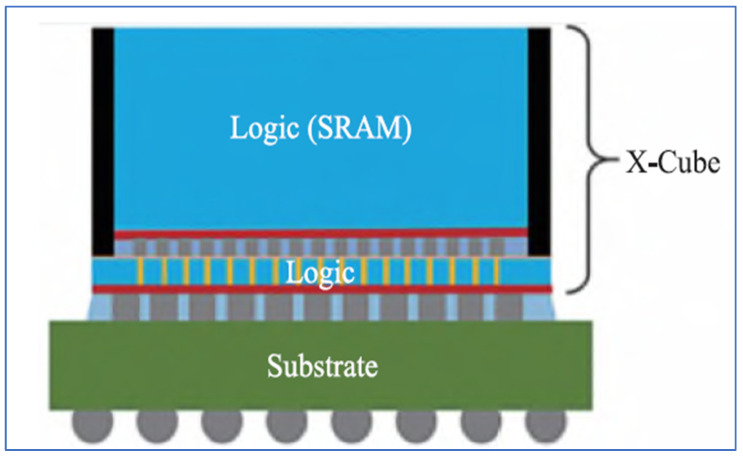
Typical 3D silicon X-Cube chiplets hetero-integration by Samsung, SK [71].

One typical example for 3D electrical chip stacking is Intel Foveros, in which various IP chips with different types of processes are directly stacked together with TSVs and bumps as shown in Figure 11. It needs no complex design and speeds up the time to market significantly. Foveros has been applied on a MAX series GPU and this is the first time Intel have deployed a supercomputing GPU in machine learning and AI. The other is Samsung’s X-Cube as shown in Figure 12. X-Cube is designed to provide high-performance integration for complex semiconductor applications. It utilizes a cutting-edge 3D stacking architecture to combine multiple logic and memory chips into a single compact package. The X-Cube technology incorporates an advanced interposer and vertical interconnects, offering enhanced bandwidth, reduced power consumption, and improved thermal management. By stacking multiple chips and efficiently interconnecting them, X-Cube enables superior performance for applications requiring high computational power, such as artificial intelligence, data centers, and high-performance computing. The technology is optimized for scalability, allowing for greater integration density and supporting heterogeneous integration, where different types of chips (e.g., CPUs, GPUs, and memory) can work together in a unified system. Samsung’s X-Cube represents a leap forward in the 3D packaging domain, offering innovative solutions for next-generation computing and communications technologies [83,84,85].

Intel’s Foveros and Samsung’s X-Cube are both advanced 3D packaging technologies designed to integrate multiple chips in a compact form for improved performance. Foveros uses a chip-stacking approach with a focus on interconnecting logic and memory vertically, enhancing bandwidth and power efficiency. It allows for heterogeneous integration, combining different types of chips, like CPUs and memory. Samsung’s X-Cube also employs 3D stacking and vertical interconnects but focuses on optimizing thermal management, reducing power consumption, and supporting high-performance applications such as AI and data centers. Both technologies aim for higher performance and density, but X-Cube emphasizes thermal optimization more than Foveros.

The 3D CPO technique is an advanced packaging technology that integrates optical components, such as lasers, photodetectors, and modulators, directly within the same package as the electronic chips in a 3D stacked configuration. This integration enables high-speed data transmission using optical interconnects while keeping the electronics and optics tightly coupled on the same substrate or interposer. The 3D stacking allows for a compact form factor with minimal signal loss and power consumption, as the optical and electronic components communicate through short, direct connections. By co-packaging optics and electronics, CPO eliminates the need for external optical-to-electrical conversions, improving efficiency and bandwidth, and addressing challenges in high-performance applications like data centers, telecommunications, and AI systems, where large amounts of data need to be transmitted quickly and efficiently. This technology is crucial for next-generation high-speed optical interconnects, reducing the latency and power consumption of inter-chip communication in large-scale systems.

**Figure 13:**
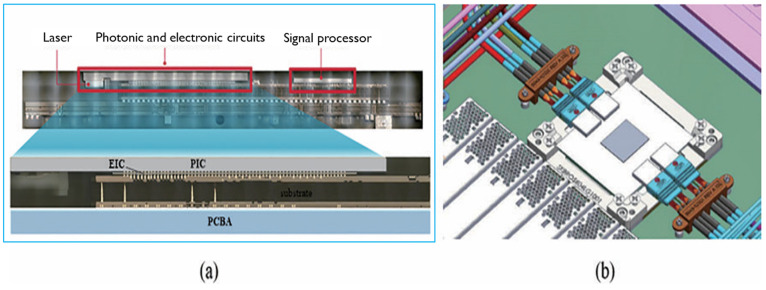
Broadcom Si 3D CPO schematic. (**a**) Cross-section of the 3D package; (**b**) schematic diagram of packaging scheme [61].

An example is Broadcom 3D Silicon CPO as shown in Figure 13. PIC is flip-chip above EIC which is connected to switch ASIC through the substrate. In this application, four CPO packages in total are adopted, which can support 12.8 Tb/s bandwidth. Power and cost are reduced by 40% with this configuration.

For 3D glass CPO integration, Intel has announced it for the next generation of high-power processors, which is very promising. Intel is on the path to delivering 1 trillion transistors on a package by 2030, and its ongoing innovation in advanced packaging including glass substrates will help achieve this goal [86]. As shown in Ref. [86] Chapter 6 Figure 6.25, the EIC, PIC, and glass are stacked vertically with switch ASIC on the side and an on-chip glass waveguide connected to off-package optical fibers.

Table 2 shows a comprehensive performances comparison of various advanced packaging technologies.

## 4. Future Prospects

With the continuous development of micro–nano electronics, Moore’s Law has encountered its physical limits; thus, semiconductor industry has turned to advanced packaging technology to solve the bottleneck problems as we discussed in the previous sections. Till today plenty of achievements have been acquired, many issues and challenges still remain such as electronic–photonic co-simulation, advanced fabrication technology, reliability of TSV, TGV, RDL, and yield issue etc. Hopefully, our future research will be helpful to address these concerns.

Our future endeavor will focus on glass-based 2.5D and 3D advanced chiplets, CPO, and also other related fields, such as photonic integrated circuits (PICs) and quantum chips, with femtosecond laser source technology. Femtosecond lasers are widely used for their ability to interact non-linearly with matter, creating novel applications in areas such as multiphoton microscopy, high-precision micro-machining, and material processing. Their ability to process various materials, including metals, glass, polymers, and biological tissues, makes them versatile tools across many industries. The minimal heat-affected zone and the high precision of femtosecond lasers allow for the creation of intricate structures with extremely fine detail, often at the micron or sub-micron level. However, these lasers require specialized equipment, and the technology tends to be more expensive and complex to operate compared to traditional laser systems. Despite these challenges, femtosecond lasers continue to be a key technology in research, medical, and industrial applications where precision and minimal thermal impact are critical. A glass chiplets package and CPO especially demand those characteristics [87,88].

With their superior performances as shown in Table 3, glass-based CPO products are very promising for being employed in the near future hyperscale datacenter with a huge amount of CPUs and GPUs, which are essential for a modern HPC and AI system. Glass-based Co-Packaged Optics (CPO) represents a transformative shift in data center networking, hopefully offering solutions to the ever-growing need for speed, bandwidth, and energy efficiency. By integrating optical and electrical components directly on a glass substrate, this technology is poised to significantly enhance the performance and scalability of data centers, supporting the next generation of high-performance computing, AI, and cloud-based applications. While challenges in cost and manufacturing remain, the long-term benefits for datacenter operators are compelling, especially as data traffic continues to increase and demands for ultra-low latency and high-speed connections rise which cannot be satisfied by Moore’s Law and silicon-based CPO technology as mentioned previously.

Other important glass-based applications and products are PICs and quantum ICs, which are the key components for future super-high-speed optical communication and quantum information technology. The production of integrated photonic circuits is a mature and expansive field, often utilizing CMOS-compatible processes to benefit from the existing micro and nano electronics facilities, enabling cost-effective mass production in materials like silicon or silicon nitride. However, actually, all passive devices in silicon photonics can be implemented on glass material, which can expect to achieve much lower propagation loss compared to silicon or silicon nitride [89,90]. Quantum technologies aim at revolutionizing how we process and communicate data, among which building a scalable quantum computing system is considered the ultimate goal. This is driven by the potential to solve complex problems, like simulating specific chemical reactions for pharmaceutical development and sampling from the distribution probability of numerous identical bosons passing through a multimode linear interferometer, a challenge known as boson sampling that current computers cannot handle. A quantum computing chip can be implemented by a unitary transfer matrix between the input and output photonic modes, reprogrammable by the user even at runtime. The matrix is designed to implement an arbitrary optical operation with thermo-optic phase shifters which can be fabricated on the glass substrate as optical waveguides with an fs laser source. As quantum technologies advance, glass-based quantum chips are likely to play an essential role in the development of more robust and scalable quantum computers. The ability to integrate glass optics with other quantum components—such as superconducting qubits or trapped ions—could lead to hybrid systems that combine the advantages of multiple quantum technologies. With ongoing advancements in quantum materials, fabrication techniques, and error correction algorithms, glass-based quantum chips have the potential to revolutionize not only computing but also communications, cryptography, and other fields of science and technology [91,92].

**Figure 14:**
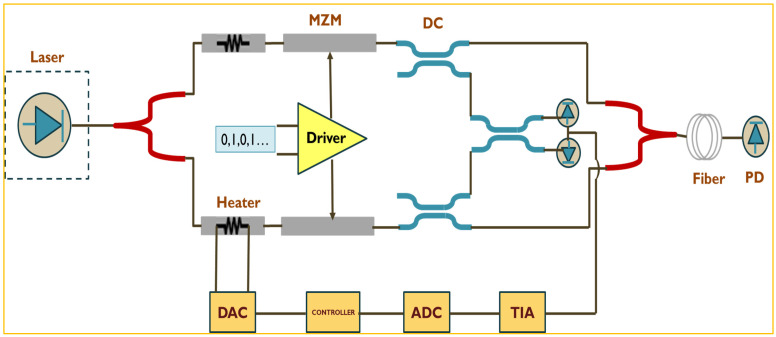
Typical link simulation setup for opto-electro co-simulation.

Next, we discuss some key challenges for glass-based technology. From the design perspective, the co-simulation of electronics and photonics is the key element to CPO mass production. There are many published results for electro-optical co-simulation from academia. Many commercial EDA software such as Cadence and Ansys Lumerical are also available for efficient simulation. In our future work, we will conduct electronic–photonic co-simulation based on a glass interposer with a glass waveguide array and copper fine-line routing. Photonic devices will be modeled by Verilog-A or SPICE language, which can be simulated together with electrical circuits. The typical link simulation setup will be as shown in Figure 14 [93,94].

**Figure 15:**
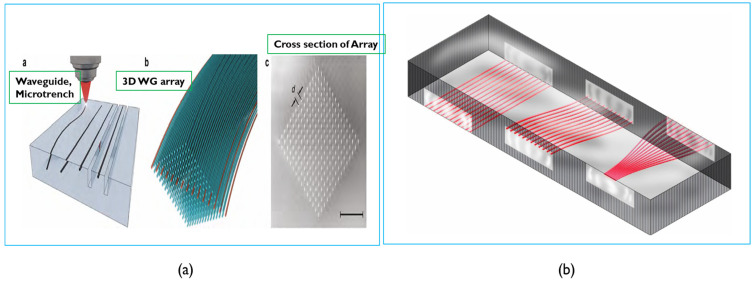
(**a**) An example of 3D glass waveguide array. (**b**) An example of planar waveguide array [95,96,97].

**Figure 16:**
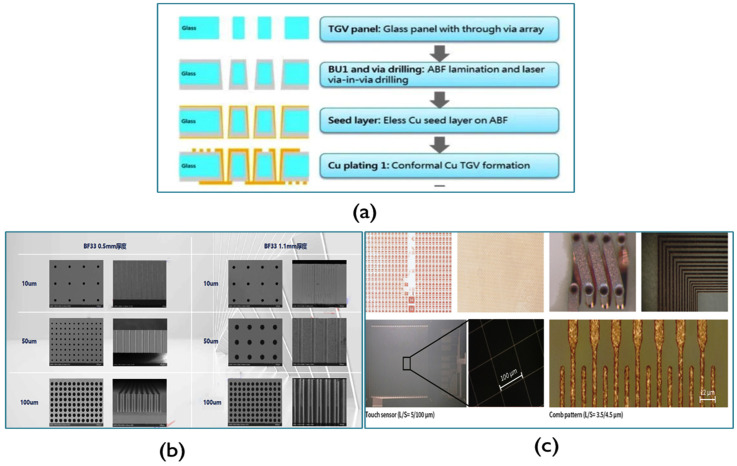
(**a**) An example of TGV structuring and metallization process flow. (**b**) Some typical samples (TGV) fabricated by fs laser. (**c**) Some typical samples (metal lines) fabricated by Cu plating [98].

From the processing perspective, fabricating high-quality, precisely mode controllable optical waveguide and through-glass vias (TGV) is the key factor for successful application in CPO and PICs and QICs. In our future work, we will adopt a femtosecond laser source to fabricate TGVs and fine-line routing directly on the glass surface and also in the bulk as electrical interconnection lines. To make optical interconnection and devices, we will also employ femtosecond laser technology to directly write in glass to generate a high-density and high-quality glass optical waveguide and other related structures. Another way to make an optical waveguide is ion exchange (IOX) as mentioned in Ref. [30]. Some glass waveguide array samples are shown in Figure 15 [95,96,97] and TGV and metal lines process flow and samples are provided in Figure 16 [98].

## 5. Concluding Remark

Entering the post-Moore era, with the rapid growth of supercomputing, AI, and hyperscale data communication, advanced packaging technologies have been considered the most significant method to further improve multi-chip-system performance in the semiconductor industry. For the last decades, important progress has been achieved; in the meantime, challenges still remain in the field such as electronic–photonic co-simulation, advanced fabrication technology, reliability issues, and power consumption, etc. In this paper, we review both conventional and advanced packaging technologies, and point out future endeavor directions based on a glass substrate, which is a superior material intended to enhance overall system performance with promising applications in chiplets, CPO, and other related fields like PIC and QIC.

## Figures and Tables

**Table 1:** Performances comparison of conventional packaging technology.

Parameter	DIP	SMT	BGA	Flip-Chip	Optical Module
I/O Density	Low (~64 pins)	Moderate	High (1000+ pins)	Very High	Moderate (fiber)
Thermal Performance	Poor	Moderate	Good	Requires underfill	Critical
Assembly Cost	Low	Low-Moderate	Moderate-High	High	Very High
Applications	Legacy systems	Consumer devices	HPC, GPUs	5G, AI chips	Data centers
Reliability	Moderate	High	High	High (with underfill)	Moderate-High

**Table 2:** Performance comparisons of advanced packaging technology.

Parameter	Fan-Out	Advanced 2D	2.5D (Si/Glass)	3D (Si/Glass)
I/O Density	500–1000 I/O/mm^2^	Up to 200 I/O/mm^2^	10 k–100 k I/O/cm^2^	10 k–1 M I/O/cm^2^
Thermal Performance	Moderate	Good	High	Critical
Cost	$–$$	$–$$	$$$	$$$$
Key Applications	Mobile, RF	CPUs, ASICs	AI/GPU, HBM	HBM, Photonics
Complexity	Moderate	Low-Moderate	High	Very High

**Table 3:** Performance comparisons of glass with silicon and organic materials.

Desired Properties		Glass	Silicon	Organic Laminate
TTV	<5 μm	Excellent	Good	Bad
Warp	<2 μm/20 mm	Excellent	Excellent	Bad
Insulation Resistance	High	Excellent	Bad	Good
Optical Transparency	Optical I/O	Excellent	Bad	Good
Surface Roughness	<5 nm	Excellent	Excellent	Bad
TCE	3.2 ppm/C	Excellent	Excellent	Bad
Hermetic Vias	Mil-Spec	Excellent	Bad	Bad
